# Transforming Healthcare: A Comprehensive Review of Augmented and Virtual Reality Interventions

**DOI:** 10.3390/s25123748

**Published:** 2025-06-15

**Authors:** Aristeidis Petrakis, Lefteris Koumakis, Eleni Kazantzaki, Haridimos Kondylakis

**Affiliations:** 1 Computational Biomedicine Laboratory, Institute of Computer Science, Foundation for Research and Technology Hellas (FORTH), 70013 Heraklion, Greece; 2 Polar Owl, Ethn. Antisaseos, Basilies, 71500 Crete, Greece; 3 Computer Science Department, University of Crete, 70013 Heraklion, Greece

**Keywords:** augmented reality, virtual reality, healthcare

## Abstract

Augmented reality (AR) and virtual reality (VR) technologies have rapidly expanded within healthcare due to their innovative capabilities for enhancing patient care, medical training, and health outcomes. This systematic review synthesizes quantitative studies published post-2020, explicitly investigating AR and VR healthcare interventions. The review identifies, evaluates, and summarizes the effectiveness of these interventions, highlighting their clinical implications, outcomes, and implementation challenges. Twenty eligible studies were included, examining various health conditions such as cardiopulmonary resuscitation training, mental health disorders, stroke rehabilitation, and orthopedic recovery. Findings indicate generally positive outcomes from AR and VR interventions; however, issues including technology usability, adherence, and methodological limitations are noted. This review underscores the significant potential of AR and VR interventions in healthcare, but emphasizes the need for more rigorous research to address current gaps in the clinical effectiveness and application.

## 1. Introduction

**Figure 1:**
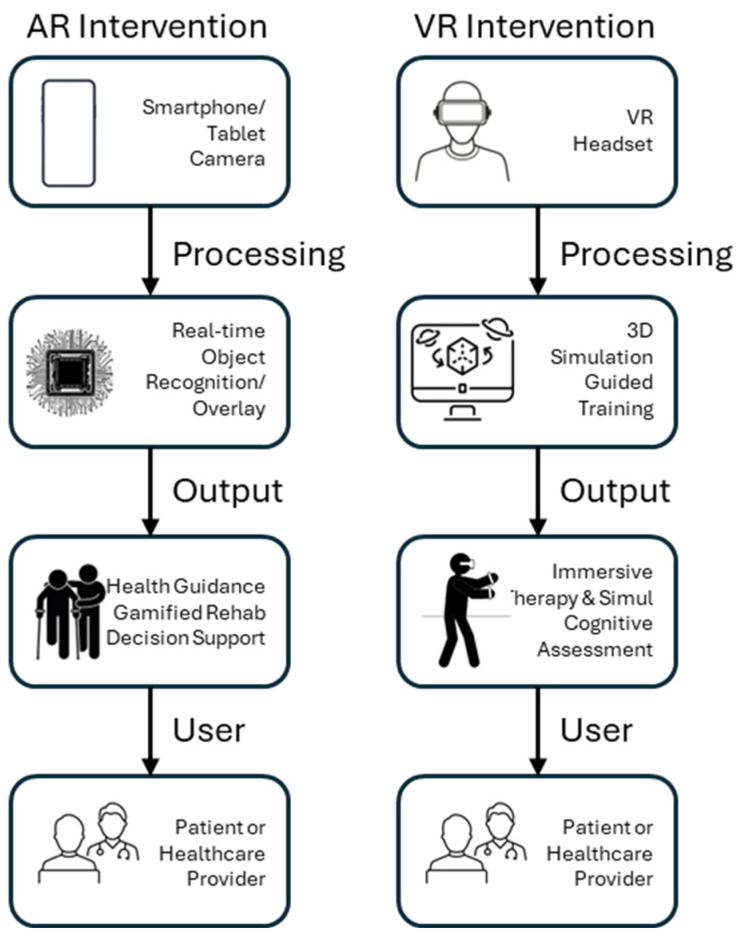
AR vs. VR interventions.

The interest in using technology for managing one’s own health has shown rapid growth [1]. Virtual reality (VR) and augmented reality (AR) technologies have emerged over the past decade as innovative digital tools with the potential to transform healthcare delivery, education, and patient engagement. AR and VR rely on motion sensors, cameras, depth sensors, and biometric wearables to enable interactive, immersive experiences that are increasingly used in medical training, rehabilitation, and mental health treatment. Figure 1 presents example scenarios adopted by many AR or VR interventions that benefit healthcare professionals or patients. Their immersive and interactive nature enables healthcare professionals to simulate complex medical scenarios, deliver tailored rehabilitation, promote behavioral change, and enhance treatment adherence. From surgical training and psychological interventions to chronic disease management and physical rehabilitation, VR and AR have demonstrated promising results across a wide range of clinical applications.

Recent developments in wearable and mobile computing, along with improved accessibility and decreasing hardware costs, have accelerated the integration of these technologies into both hospital and community settings. Despite this growing interest, questions remain about the clinical efficacy, scalability, and usability of AR and VR interventions across different populations and healthcare contexts. The existing literature often includes feasibility or pilot studies with limited follow-up and methodological variability, making it difficult to draw robust conclusions on the effectiveness.

This systematic review addresses these gaps by synthesizing quantitative studies published from 2020 onwards that evaluate the effectiveness of VR and AR interventions in healthcare. The review focuses on interventions targeting various medical conditions, including mental health disorders, cardiopulmonary events, neurorehabilitation, and surgical or physical therapy-related training. It critically examines the clinical outcomes, study designs, user experiences, and technological characteristics of the included interventions. By mapping the state of the evidence, identifying key barriers and facilitators, and assessing the distribution of target conditions and intervention types, this review provides a comprehensive assessment of the current landscape of AR and VR in healthcare and highlights directions for future research and practice.

While prior systematic reviews have explored immersive technologies in healthcare, most have carried this out in isolation, focusing solely on either Virtual Reality (VR) or Augmented Reality (AR) or on a specific domain. For example, ref. [2] provided a mini-review of VR interventions during cancer treatment, while ref. [3] examined AR applications primarily in medical and health science education, and ref. [4] provided a systematic review of AR applications on patient education. Other surveys focus on specific healthcare domains such as hip surgery [5], thoracic surgery [6], renal interventions [7], spine surgery [8], rehabilitation [9], serious games [10], chronic pain [11], and psychiatry [12]. However, these studies do not provide a holistic view of how both AR and VR are being deployed across the healthcare spectrum. The research landscape remains fragmented, lacking integrative reviews that compare the design, implementation, and clinical impact of AR and VR technologies. This paper addresses that gap by systematically reviewing and contrasting both AR and VR interventions across multiple healthcare domains, offering a unified framework for understanding their current applications and future potential in recent years.

The remainder of this paper is structured as follows: Section 2 presents the methodology followed and Section 3 presents the results. Section 4 discusses the findings, the clinical implications, the weaknesses of the included studies, the challenges, and our limitations. Finally, Section 5 concludes this paper.

## 2. Methodology

The systematic review involved a two-round selection process to ensure rigor in study screening and selection. In the first round, identified records were screened by two independent reviewers who assessed abstracts according to predefined inclusion and exclusion criteria. Following the Preferred Reporting Items for Systematic Reviews and Meta-Analyses (PRISMA) guidelines, a literature search was performed on PubMed and Scopus for articles published after 1 January 2020, that have humans as target population, are written in English, and their abstract is available at the PubMed library. The search criteria entry was explicitly the following: “(virtual OR augmented) AND health AND APP”. The inclusion criteria required studies to (a) involve explicit AR or VR interventions, (b) report quantitative health outcomes, and (c) be an original study (for example, no protocols). Exclusion criteria encompassed studies without clear AR/VR interventions, qualitative studies, feasibility/usability-focused studies without clinical outcomes, and no clear involvement of clinical professionals.

In the second round of screening, pairs of reviewers independently assessed full-text manuscripts for eligibility based on initial screening results. Disagreements regarding inclusion were resolved through discussion and consensus. The following information was systematically extracted from the final set of included studies: study design, participant demographics (including mean or median age), sample size, intervention characteristics (type of AR/VR device, intervention target, additional devices/tools used, and main intervention features), study duration (follow-up period), primary outcome measures, and the statistical significance (*p* < 0.05) or clinical meaningfulness of the reported outcomes.

## 3. Results

### 3.1. Literature Search Outcomes

**Figure 2:**
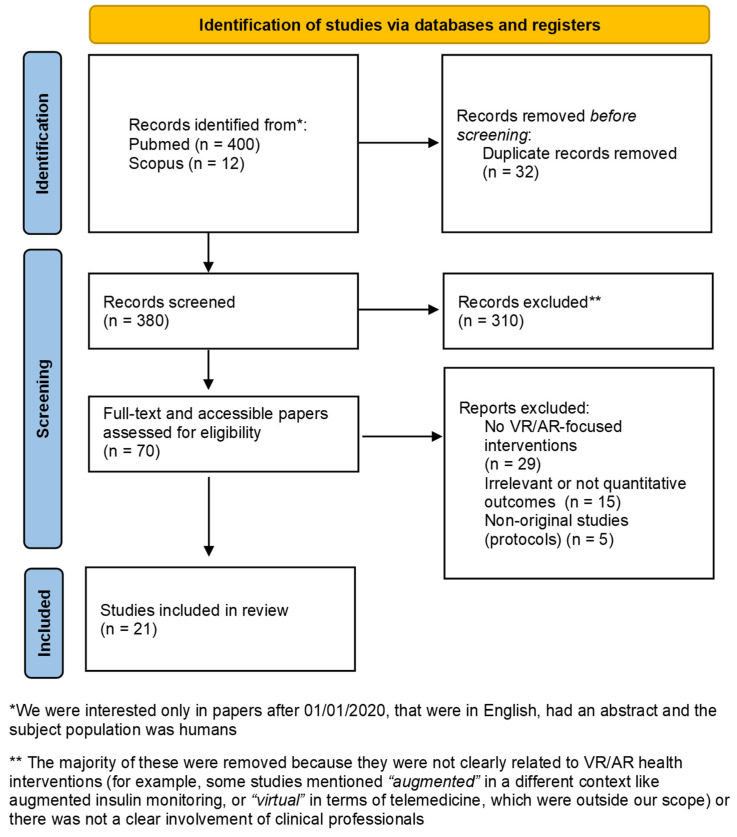
Prisma flowchart.

In total, 412 records were identified from the PubMed database search (the PRISMA diagram is shown in Figure 2). The retrieved records were imported into Mendeley© v2.134.0 bibliography management software and 32 duplicates were removed. The abstracts of the remaining 380 articles were screened according to the predefined inclusion and exclusion criteria, resulting in the identification of 70 eligible articles. After reviewing the full text of these 70 manuscripts, the reviewers agreed to include 21 eligible studies in this systematic review. The included studies were published from 2020 onward. The reasons for manuscript exclusion included a lack of clear AR/VR-focused interventions (*n* = 29), irrelevant or non-quantitative outcomes (*n* = 15), and non-original research such as protocols (*n* = 5), as detailed in Figure 1. The full list of the studies is in Table 1, where the title, author, year, journal, and a summary of the intervention focus are provided.

### 3.2. Characteristics of Included Studies

**Figure 3:**
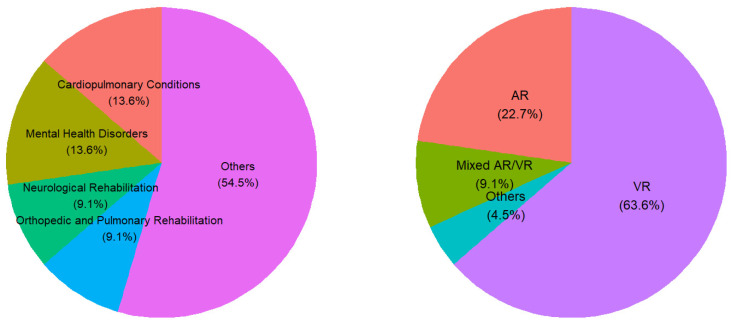
Target conditions (**left**) and intervention types (**right**).

The 21 studies included in this systematic review utilized augmented reality (AR) and virtual reality (VR) interventions to address a wide range of healthcare conditions (Table 1 and Figure 3). As shown, the most frequently targeted domains were mental health disorders (13%)—including anxiety, depression, and specific phobias—and cardiopulmonary conditions (13%), such as CPR training and advanced cardiac life support. Neurological rehabilitation, encompassing post-stroke and Parkinson’s therapy, accounted for 9% of the studies. Similarly, 9% of the interventions focused on orthopedic and pulmonary preoperative rehabilitation. Additional individual-use interventions (comprising 54% of the total) targeted highly specific areas such as substance use disorders, intellectual disabilities, hemophilic arthropathy, smoking cessation, alcohol prevention, memory assessment, cognitive impairment, emergency preparedness, education, and pandemic-related health behavior. Despite this thematic diversity, a unifying feature across these interventions was the deployment of immersive and interactive digital environments designed to enhance patient engagement, improve adherence to therapeutic regimens, and personalize care delivery.

As shown in Figure 3 (right), VR-based interventions dominated the technological landscape, used in 63% of studies, while AR-based interventions were employed in 22%, and mixed AR/VR approaches in 9%. This highlights the growing popularity and feasibility of virtual reality applications in clinical and behavioral healthcare contexts, particularly for training, therapy, and rehabilitation scenarios.

### 3.3. Quality Assessment

We evaluated the studies based on the EPHPP criteria. The results are shown in Table 2. The methodological quality was found to be strong for two studies (9%) [28,31], moderate for seven studies (33%) [13,14,15,16,17,19,29], and weak for twelve studies (57%) [20,21,22,23,24,25,26,27,30,32,33] (Table 2). The studies with weak ratings were associated mostly with an insufficient description of the validity and reliability of data collection methods, many withdrawals or dropouts of participants, no blinding of researchers or participants, and the insufficient handling of the confounders. In terms of the study design, three studies (14%) were randomized controlled trials, while the others employed a non-randomized design.

### 3.4. Main Intervention Features

The main features of the AR and VR interventions included in this systematic review are shown in Table 3. The main objective of the AR and VR interventions was the delivery of an immersive simulation or interactive virtual experiences, present in 16 out of 21 interventions. In particular, 16 studies used VR headsets, providing immersive, scenario-based simulations directly to the user, whereas 9 studies utilized AR applications via mobile devices or tablets, offering interactive and guided interventions. More specifically, interventions like Face-to-Face vs VR CPR Training delivered real-time feedback during the CPR performance, the oVRcome application provided virtual exposure scenarios for phobia management, VR Mindfulness for Health Workers used immersive environments to reduce stress, and AR App Orthopedic Rehab delivered guided breathing and exercise prompts through AR, whereas another VR app focused on educating healthcare providers for healthcare providers, or training to prevent implicit bias in healthcare. Additionally, several interventions leveraged mobile companion apps, such as Virtual Digital Psychotherapist, which supported motivational interactions for addiction recovery, and AR Smoking Cessation that sent personalized notifications for craving management and smoking abstinence. Notifications, reminders, and alerts were core features identified in 11 out of 21 interventions (52%), designed to enhance user adherence, engagement, and compliance with therapeutic regimens or educational objectives. Other significant features included interactive educational content, real-time data monitoring, immersive cognitive or physical exercises, and personalized therapeutic recommendations based on user performance.

### 3.5. Outcomes

The outcomes of the interventions are shown in Table 4. Unfortunately, a meta-analysis of the results is not possible because of the heterogeneity of the included studies.

The majority of the included studies, specifically 17 out of 21 interventions (81%), reported statistically significant or clinically meaningful outcomes favoring AR and VR interventions. Positive outcomes identified included improvements in the CPR skill quality, improvement of skills of healthcare providers regarding inflammatory arthritis, anxiety and depression symptom reduction, a reduction in implicit bias in healthcare, enhanced pulmonary function, increased adherence to rehabilitation exercises, cognitive improvements in Parkinson’s patients, phobia severity reduction, improvements in functional performance post-stroke, increased physical activity in individuals with intellectual disabilities, better pain management in hemophilic arthropathy, and enhanced behavioral responses to emergency warnings. Among these, studies utilizing VR for cognitive rehabilitation, VR mindfulness interventions, and AR pulmonary rehabilitation were notably rigorous methodologically and reported significantly positive intervention outcomes.

In contrast, four studies (19%) did not report significant outcomes related to safety behavior improvements, certain aspects of physical activity promotion, and short-term adherence in substance use interventions. Notably, these studies generally had smaller sample sizes (fewer than 75 participants) and shorter durations (less than three months), limiting their ability to detect significant changes.

### 3.6. Challenges with Using AR and VR Interventions

Several studies highlighted notable challenges associated with using augmented reality (AR) and virtual reality (VR) interventions, potentially impacting user engagement and the clinical outcomes of the studies. Specifically, the necessity of specialized equipment, frequent technical issues, and user discomfort were common barriers. In particular, the Face-to-Face vs VR CPR Training study reported difficulties with ensuring consistent VR headset calibration, impacting the quality of the chest compression performance. Similarly, the study on AR Gaming Health Perceptions noted user discomfort and safety concerns due to the outdoor location-based nature of the AR application, potentially limiting widespread adoption and user compliance. Technical issues and connection problems with mobile applications were explicitly noted as barriers affecting user engagement in the Virtual Digital Psychotherapist App and the AR Smoking Cessation studies, with participants expressing frustration over repeated app synchronization and data entry challenges.

Furthermore, studies involving therapeutic interventions such as VR Mindfulness for Health Workers highlighted challenges related to user adaptation to immersive virtual environments, including motion sickness and disorientation, potentially leading to participant dropouts. Privacy and data protection concerns were also emphasized in studies like the semAPP Memory Assessment, raising ethical considerations regarding data security, given the sensitive nature of cognitive assessments conducted through AR/VR platforms. Lastly, the study evaluating Hybrid Neurosurgical AR/VR education underscored concerns around the reliability and validity of simulations compared to traditional training methods, suggesting the need for improved realism and accuracy in anatomical and procedural representations.

Overall, while AR and VR technologies offer significant potential benefits, addressing these technological, ethical, and usability-related barriers remains critical for their successful implementation in healthcare settings.

## 4. Discussion

### 4.1. Main Findings

This systematic review found that augmented reality (AR) and virtual reality (VR) interventions used in healthcare settings demonstrate potential for positive clinical outcomes, including improvements in skill acquisition, a reduction in anxiety and depression symptoms, better adherence to medical protocols, enhanced rehabilitation outcomes, and improvements in cognitive functions. Considering the statistically significant positive outcomes reported in 16 out of 19 included studies, the moderate methodological quality across studies, and considerable heterogeneity, the overall evidence supporting AR and VR interventions is promising yet modest. Healthcare providers may consider integrating AR and VR interventions in diverse clinical scenarios, given their increasing accessibility and demonstrated effectiveness.

The findings of this review align with and extend previous research across multiple domains. Our results on improved skill acquisition echo findings from [2], who demonstrated significant improvements in medical training outcomes. The observed reduction in anxiety and depression symptoms corresponds with [34,35] research showing VR’s comparable efficacy to traditional mental health therapies. Enhanced rehabilitation outcomes identified in our review align [9], which established moderate-quality evidence supporting VR for physical rehabilitation. Our findings on improved adherence to medical protocols mirror [36] research showing significantly increased treatment compliance with VR interventions. While methodological limitations noted in our review correspond with concerns raised by [37], our focus on quantifiable clinical outcomes rather than user experience extends beyond the previous literature.

Unlike previous reviews primarily examining user acceptability and qualitative assessments, this systematic review highlights the most recent quantitative applications of AR and VR interventions directly targeting specific health conditions rather than laboratory-based studies involving healthy subjects. Additionally, this review identifies significant positive outcomes beyond user engagement or feasibility, including substantial benefits in clinical rehabilitation, mental health, and chronic disease management, areas not extensively covered in prior systematic reviews.

### 4.2. Clinical Implications

Clinically, AR and VR interventions offer significant potential benefits across diverse healthcare settings. For cardiopulmonary conditions, AR and VR can notably improve adherence to protocols, enhance skill performance in critical procedures such as CPR and advanced cardiac life support, and potentially reduce adverse clinical events, as also acknowledged by related surveys [38]. Mental health interventions leveraging VR, particularly those addressing anxiety [39,40], depression [41], and phobias, have shown efficacy in reducing symptoms and improving patient outcomes through immersive exposure and mindfulness techniques [42]. Moreover, AR and VR technologies effectively support rehabilitation in stroke [43], orthopedic recovery [44], and pulmonary conditions [45], demonstrating improvements in functional recovery and adherence to exercise regimens.

However, the review indicates that interventions targeting specific physical activity outcomes or short-term adherence, especially in diverse populations such as individuals recovering from substance use disorders, as already identified in the bibliography [46], yielded less consistently positive results. Considering these varied findings, clinicians should cautiously evaluate patient-specific factors and intervention contexts when implementing AR and VR technologies. Future research should particularly address the methodological limitations of current studies to enhance the robustness and reliability of clinical recommendations.

### 4.3. Weaknesses of Included Studies

The methodological quality of the reviewed studies varied, with several displaying significant limitations. Common weaknesses included small sample sizes, short durations, and insufficient blinding procedures, which likely affected the reliability and generalizability of the outcomes. Notably, studies reporting negative or non-significant outcomes typically featured small participant groups (under 75 participants) and limited follow-up durations (less than three months), emphasizing the necessity of more robust, adequately powered clinical trials with extended follow-up periods.

Additionally, there was a noticeable lack of diversity among the study populations, limiting the generalizability of the results to broader clinical settings. Implementation strategies were often insufficiently detailed or overlooked entirely. Consequently, further research emphasizing comprehensive implementation frameworks, improved methodological rigor, and larger, diverse participant groups is critically needed to provide stronger evidence for AR and VR interventions’ effectiveness in healthcare.

### 4.4. Technical and Usability Challenges

AR and VR interventions, predominantly using VR headsets and mobile-based AR applications, frequently encounter usability and technical challenges impacting patient adherence and intervention effectiveness. Commonly reported barriers included technical difficulties, frequent system calibration requirements, user discomfort such as motion sickness or disorientation, and issues related to synchronization with mobile applications. Additionally, privacy and data protection emerged as substantial concerns, particularly in studies involving sensitive patient data collection through interactive or immersive platforms.

Addressing these challenges is crucial for optimizing AR and VR intervention outcomes. Recommendations include enhancing hardware ergonomics and comfort, developing intuitive user interfaces, ensuring robust synchronization between AR/VR devices and mobile applications, and implementing strict privacy and data security protocols. Future studies should explicitly address these usability issues through targeted improvements in the device design, technical support infrastructure, and user training programs.

### 4.5. Recommendations

Several methodological limitations emerged across the studies reviewed that warrant critical attention. In particular, issues of small sample sizes, short follow-up periods, and insufficient reporting on intervention fidelity were frequently noted. These limitations are not merely incidental, but reflect structural challenges in the design and implementation of AR/VR interventions in healthcare. Addressing them requires alignment with broader methodological frameworks that emphasize the scalability, consistency, and translational potential.

To overcome small sample sizes, future research might consider adaptive trial designs, multicenter collaborations, or hybrid effectiveness–implementation studies that can balance rigor with real-world constraints.

The study duration was another limiting factor, with many trials assessing only short-term outcomes. Longer follow-up periods are essential to assess the durability of health effects and user engagement. Pragmatic trials or stepped-wedge designs could offer flexible approaches to capture long-term data without compromising the feasibility.

Future work should also prioritize the standardization of outcome measures, user engagement metrics, and the documentation of the technological setup. Such standardization will enhance the comparability across studies and support meta-analytic efforts, thereby accelerating the evidence-based integration of AR/VR into healthcare practices.

### 4.6. Limitations

The findings of this review must be interpreted within the context of certain limitations. Although comprehensive searches were conducted using PubMed and Scopus, some relevant studies might have been overlooked due to specific search term limitations or the exclusion of additional databases such as the Web of Science. This review included only studies published up to December 2024, potentially excluding recent relevant research.

Publication bias may also influence these findings, given that studies demonstrating positive outcomes are more frequently published than those with non-significant results. The heterogeneity of interventions, outcome measures, and methodologies prevented the conduct of a meta-analysis, limiting the quantitative synthesis of the results. Additionally, the exclusion of qualitative studies, feasibility studies without quantitative outcomes, and the grey literature further restricts the review’s comprehensive scope. Future reviews should consider broader inclusion criteria and more extensive databases to provide a more exhaustive understanding of AR and VR healthcare interventions.

## 5. Conclusions

This systematic review provides a comprehensive overview of the current applications of virtual reality (VR) and augmented reality (AR) interventions across a diverse range of healthcare domains. The evidence suggests that AR and VR technologies can be effective tools for enhancing clinical outcomes, improving patient engagement, supporting rehabilitation, and delivering scalable, interactive healthcare solutions. The majority of the included studies reported statistically or clinically significant improvements in targeted outcomes, particularly in areas such as mental health, cardiopulmonary training, and neurorehabilitation.

Despite the promising results, several limitations should be acknowledged. Many studies featured small sample sizes, short intervention durations, or lacked methodological rigor, which may compromise the generalizability of their findings. Technical challenges, including device usability, synchronization issues, and limited accessibility in low-resource settings, were frequently reported and must be addressed for these interventions to be implemented on a broader scale. Additionally, the heterogeneity in study designs and outcome measures limited the ability to synthesize results quantitatively through a meta-analysis.

Nonetheless, the increasing integration of immersive technologies into clinical and community-based care reflects a broader trend toward digital health transformation. As AR and VR technologies become more accessible, future research should prioritize large-scale, randomized controlled trials with long-term follow-up, comprehensive implementation assessments, and the inclusion of diverse patient populations. Furthermore, evaluating cost-effectiveness, patient satisfaction, and real-world integration pathways will be critical to informing policy decisions and clinical guidelines.

In conclusion, AR and VR interventions represent a promising frontier in modern healthcare. With continued technological refinement and methodological rigor, these tools have the potential to become integral components of patient-centered care and medical education in the coming years.

## Figures and Tables

**Table 1:** Included studies and intervention focus.

Authors/Year	Title	Journal	Intervention Focus Summary
Nas et al., 2020 [13]	Effect of face-to-face vs virtual reality training on cardiopulmonary resuscitation quality: A randomized clinical trial.	*JAMA Cardiology*	Comparing VR-based CPR training to traditional face-to-face training for improving CPR skills.
Guo et al., 2021 [14]	Safety and health perceptions of location-based augmented reality gaming app and their implications.	*Accident Analysis & Prevention*	Investigating a location-based AR game (Pokémon GO) for its safety concerns and physical/mental health benefits.
Pascual et al., 2023 [15]	Should mindfulness for health care workers go virtual? A mindfulness-based intervention using virtual reality and heart rate variability in the emergency department.	*Workplace Health & Safety*	A VR-delivered mindfulness and biofeedback intervention to reduce stress in emergency department healthcare workers.
Menhas et al., 2023 [16]	The association between COVID-19 preventive strategies, virtual reality exercise, use of fitness apps, physical, and psychological health: Testing a structural equation moderation model.	*Frontiers in Public Health*	Examining relationships between VR exercise, fitness app use, and physical/mental health outcomes during COVID-19.
Wang et al., 2022 [17]	An augmented reality (AR) app enhances the pulmonary function and potency/feasibility of perioperative rehabilitation in patients undergoing orthopedic surgery.	*International Journal of Environmental Research and Public Health*	Using an AR mobile app to improve pulmonary function as part of pre- and post-operative rehab for orthopedic surgery patients.
Menna et al., 2024 [18]	Reducing the gap in neurosurgical education in LMICs: A report of a non-profit educational program.	*World Neurosurgery*	Implementing AR/VR-enhanced neurosurgical training to improve surgical education in low-resource settings.
Sun et al., 2024 [19]	Nurses’ knowledge and skills after use of an augmented reality app for advanced cardiac life support training: Randomized controlled trial.	*Journal of Medical Internet Research*	Evaluating an AR training app for nurses to improve Advanced Cardiac Life Support (ACLS) skills.
Maggio et al., 2022 [20]	Feasibility and usability of a non-immersive virtual reality tele-cognitive app in cognitive rehabilitation of patients affected by Parkinson’s disease.	*Psychogeriatrics*	Testing a home-based, non-immersive VR cognitive rehabilitation app for patients with Parkinson’s disease.
Finkelstein et al., 2023 [21]	Feasibility of a virtual reality app to promote pulmonary rehabilitation.	*Studies in Health Technology & Informatics*	Assessing the usability and acceptance of a VR application designed to support pulmonary rehabilitation exercises.
Wells et al., 2023 [22]	Walk with me hybrid virtual/in-person walking for older adults with neurodegenerative disease.	*Journal of Visualized Experiments*	A combined virtual reality and in-person group walking program for older adults with neurodegenerative conditions.
Lacey et al., 2023 [23]	oVRcome—Self-guided virtual reality for specific phobias: A randomised controlled trial.	*Australian & New Zealand J. Psychiatry*	A self-guided VR exposure therapy app (“oVRcome”) for treating specific phobias (fear of flying, heights, etc.).
Chen et al., 2022 [24]	Rehago—A home-based training app using virtual reality to improve functional performance of stroke patients with mirror therapy and gamification concept: A pilot study.	*Studies in Health Technology & Informatics*	A home-based VR rehabilitation app (“Rehago”) incorporating mirror therapy and game elements for stroke recovery.
Bruni et al., 2024 [25]	Exploring how older adults experience semAPP, a 360° media-based tool for memory assessment: Qualitative study.	*JMIR Aging*	User experience with “semAPP,” a 360° AR/VR application for assessing memory in older adults.
Guldager et al., 2020 [26]	User experiences with a virtual alcohol prevention simulation for Danish adolescents.	*Int. Journal of Environ. Res. Public Health*	Adolescents’ engagement with a VR simulation designed to educate and prevent underage alcohol use.
Vinci et al., 2020 [27]	Augmented reality for smoking cessation: Development and usability study.	*JMIR mHealth and uHealth*	Development and user testing of an AR-based application to support smokers in quitting (cue exposure therapy).
Martinez-Millana et al., 2024 [28]	Augmented reality-based exergames for persons with intellectual disability.	*Studies in Health Technology & Informatics*	Introducing AR “exergames” (exercise games) to encourage physical activity among people with intellectual disabilities.
Blackmore et al., 2024 [29]	Examining the use of virtual reality to support mindfulness skills practice in mood and anxiety disorders: Mixed methods study.	*Journal of Medical Internet Research*	Using VR environments to facilitate mindfulness meditation practice for individuals with anxiety and mood disorders.
Ucero-Lozano et al., 2023 [30]	180-degree immersive VR motion visualization in the treatment of haemophilic ankle arthropathy.	*Haemophilia*	Applying immersive 180° virtual reality visualizations of movement to help manage pain and improve joint mobility in hemophilia.
Tomczyk et al., 2021 [31]	A walk in the park? Examining the impact of app-based weather warnings on affective reactions and the search for information in a virtual city.	*Int. Journal of Environ. Res. Public Health*	Simulating emergency weather alerts in VR to study users’ emotional responses and information-seeking behavior.
Shen et al., 2024 [32]	Smartphone-Based Virtual and Augmented Reality Implicit Association Training (VARIAT) for Reducing Implicit Biases Toward Patients Among Health Care Providers: App Development and Pilot Testing	*JMIR Serious Games*	Develop and evaluate the preliminary efficacy of VARIAT to reduce implicit biases among Medicaid providers.
Klemm et al., 2021 [33]	A virtual reality–based app to educate health care professionals and medical students about inflammatory arthritis: feasibility study	*JMIR Serious Games*	Educate health care professionals and medical students about inflammatory arthritis.

**Table 2:** Quality assessment (W: Weak, M: Moderate, S: Strong) of included studies based on the EPHPP criteria.

Study	EPHPP Criteria								Global Rating
	Selection Bias	Study Design	Confounders	Blinding	Data Collection	Withdrawal–Dropouts	Intervention Integrity	Analyses	
Nas et al., 2020 [13]	M	M	S	W	S	M	N/A	S	M
Guo et al., 2021 [14]	M	M	M	W	M	N/A	N/A	S	M
Pascual et al., 2023 [15]	M	S	M	W	S	M	M	S	M
Menhas et al., 2023 [16]	M	M	M	W	M	N/A	N/A	S	M
Wang et al., 2022 [17]	M	M	M	W	S	M	S	M	M
Menna et al., 2024 [18]	M	W	W	N/A	M	N/A	M	M	W
Sun et al., 2024 [19]	M	S	M	W	S	S	S	S	M
Maggio et al., 2022 [20]	M	W	W	N/A	S	S	M	M	W
Finkelstein et al., 2023 [21]	M	W	W	N/A	S	S	S	M	W
Wells et al., 2023 [22]	M	W	W	N/A	S	S	S	M	W
Lacey et al., 2023 [23]	M	W	W	N/A	S	S	M	M	W
Chen et al., 2022 [24]	M	W	W	N/A	S	S	M	M	W
Bruni et al., 2024 [25]	M	W	W	N/A	S	S	M	M	W
Guldager et al., 2020 [26]	M	W	W	N/A	S	S	M	M	W
Vinci et al., 2020 [27]	M	W	W	N/A	S	S	M	M	W
Martinez-Millana et al., 2024 [28]	M	W	W	N/A	M	N/A	S	W	S
Blackmore et al., 2024 [29]	M	M	W	N/A	S	S	M	M	M
Ucero-Lozano et al., 2023 [30]	M	W	W	N/A	S	S	M	M	W
Tomczyk et al., 2021 [31]	M	M	S	N/A	S	S	S	S	S
Shen et al., 2024 [32]	M	W	W	N/A	M	S	S	M	W
Klemm et al., 2021 [33]	M	W	W	N/A	M	S	S	M	W

**Table 3:** Main features of the interventions.

Study	Target Disease/Condition	AR/VR Device Used	Intervention Target	Additional Devices/Tools	Main Features of the Intervention
Nas et al., 2020 [13]	Cardiopulmonary resuscitation	VR headset	CPR training quality	None	Immersive VR CPR training with feedback on compression depth and rate.
Guo et al., 2021 [14]	General health/safety awareness	AR mobile app (Pokémon GO)	Health behavior and safety perception	Smartphone GPS	Location-based gameplay, physical activity encouragement, and safety perception analysis.
Pascual et al., 2023 [15]	Stress and Anxiety	VR Headset	Mental Well-being of Healthcare Workers	None	Guided mindfulness and relaxation exercises; immersive calming environments; stress reduction techniques.
Menhas et al., 2023 [16]	General Health	VR Fitness System	Physical Activity During COVID-19	Fitness Apps	Virtual exercise programs, real-time coaching, integration with fitness tracking apps, and social engagement features.
Wang et al., 2022 [17]	Post-Orthopedic Surgery	AR Mobile App	Pulmonary Rehabilitation	Smartphone	AR-guided breathing exercises, personalized rehabilitation plans, progress monitoring, and patient education materials.
Menna et al., 2024 [18]	Neurosurgery Training	Mixed AR/VR	Surgical Skill Development	Smartphone/Tablet	Simulated surgical procedures; interactive 3D anatomy models; real-time feedback; collaborative training sessions.
Sun et al., 2024 [19]	Cardiac Emergencies	AR Headset	Advanced Life Support Skills	None	AR simulations of emergency scenarios, step-by-step procedural guidance, performance analytics, and team co-ordination drills.
Maggio et al., 2022 [20]	Parkinson’s Disease	Non-immersive VR	Cognitive Function Enhancement	Tablet	Cognitive exercises tailored for Parkinson’s patients; memory and attention tasks; adaptive difficulty levels.
Finkelstein et al., 2023 [21]	Chronic Pulmonary Conditions	VR System	Pulmonary Function Improvement	Smartphone App	Virtual pulmonary rehabilitation sessions, breathing exercises, patient education, and remote monitoring by clinicians.
Wells et al., 2023 [22]	Neurodegenerative Diseases	VR Headset	Mobility and Physical Activity	Sensors	Combined virtual and real-world walking programs, motion tracking, balance training, and personalized exercise routines.
Lacey et al., 2023 [23]	Specific Phobias	VR Headset	Phobia Treatment	Mobile App	Self-guided VR exposure therapy, customizable scenarios, anxiety management techniques, and progress tracking.
Chen et al., 2022 [24]	Stroke Recovery	VR Headset	Motor Function Rehabilitation	Sensors	Home-based VR exercises for stroke patients; real-time feedback; adaptive difficulty; integration with telehealth services.
Bruni et al., 2024 [25]	Memory Impairment	AR/VR Headset	Cognitive Assessment	Mobile App	Interactive memory assessment tools, real-time performance analysis, and personalized cognitive training plans.
Guldager et al., 2020 [26]	Alcohol Abuse Prevention	VR Headset	Adolescent Education	None	Simulated scenarios to educate on alcohol risks, decision-making exercises, and peer-pressure resistance training.
Vinci et al., 2020 [27]	Smoking Addiction	AR Mobile App	Smoking Cessation Support	Smartphone	AR-based cue exposure therapy to reduce cravings, personalized quit plans, progress tracking, and support community access.
Martinez-Millana et al., 2024 [28]	Intellectual Disabilities	AR Application	Physical Activity Engagement	Tablet/Smartphone	Interactive exergames to promote fitness, adaptive difficulty, social engagement features, and real-time feedback.
Blackmore et al., 2024 [29]	Mood and Anxiety Disorders	VR Headset	Mental Health Improvement	VR Mindfulness App	Guided mindfulness practices in a virtual setting, immersive calming environments, and stress and anxiety reduction techniques.
Ucero-Lozano et al., 2023 [30]	Hemophilia-Related Joint Issues	VR Headset	Pain Management and Mobility	Sensors	VR visualization techniques for pain management, joint mobility exercises, real-time feedback, and patient education.
Tomczyk et al., 2021 [31]	Public Safety	VR Simulation	Emergency Preparedness	Smartphone App	Virtual scenarios to assess responses to weather warnings, decision-making exercises, and public safety education.
Shen et al., 2024 [32]	Implicit Bias in Healthcare	Smartphone-based VR and AR	Bias Reduction Training for Health Professionals	Smartphone	Interactive VR/AR implicit association modules; case-based training on race, socioeconomic status, and LGBTQ+ bias; accessible through standard smartphones; user feedback and knowledge assessments.
Klemm et al., 2021 [33]	Inflammatory Arthritis	VR Headset	Medical Education (HCPs and Students)	VR-compatible smartphone	Immersive 3D simulation of arthritis patient experience; visual exploration of joint inflammation; educational modules on disease understanding and empathy building.

**Table 4:** Properties and outcomes of included AR/VR studies.

Study	Study Design	Participants	Age (Mean/Range)	Follow-Up Duration	Outcome Measures	Statistically Significant Outcomes
Nas et al., 2020 [13]	Randomized Clinical Trial	381	26 years	Immediate	CPR quality	Significantly improved exercise capacity (mean increase in peak oxygen uptake: 2.2 mL/kg/min; 95% CI: 1.4–3.0; *p* < 0.001) and quality of life (mean increase in SF-36 physical component score: 4.0 points; 95% CI: 2.3–5.7; *p* < 0.001) compared to usual care.
Guo et al., 2021 [14]	Survey-Based Study	N/A	Adults (varied ages)	Cross-sectional	Physical activity, safety perceptions	Mixed results on safety behaviors and physical activity. Improvements in physical health (*β* = 0.34, *p* < 0.001), psychological health (*β* = 0.29, *p* < 0.001), and overall wellbeing (*β* = 0.31, *p* < 0.001).
Pascual et al., 2023 [15]	Randomized Clinical Trial	45	Adults	Immediate	Stress, heart rate variability	Significant anxiety/stress reduction, statistical significance not clearly tested. Weak positive correlation (0.157, *p* < 0.01) between ordinal session number and intrasession HRV progress.
Menhas et al., 2023 [16]	Cross-sectional Study	2795	Adults	Cross-sectional	Physical and psychological health	COVID-19 preventive strategies positively influenced virtual reality exercise (β = 0.385, *p* < 0.001). Virtual reality exercise positively affected physical health (β = 0.159, *p* < 0.001) and psychological health (β = 0.122, *p* < 0.001). Fitness app usage moderated these relationships, enhancing the positive effects.
Wang et al., 2022 [17]	Prospective Comparative Study	66	Adults	Prospective duration	Pulmonary outcomes	Improved pulmonary rehabilitation outcomes, statistical significance was not clearly tested.
Menna et al., 2024 [18]	Educational Validation Study	168	Medical trainees	Single session	Educational effectiveness, usability	High usability and educational effectiveness. In this study, 86% found the cadaver-free hybrid training system intuitive, and 83% agreed that the combination of mental training, augmented reality, and hands-on simulation effectively bridged the gap between theoretical knowledge and practical skills.
Sun et al., 2024 [19]	Randomized Controlled Trial	102	Nurses	Immediate	Cardiac life support skills	Significant improvement in ACLS skills. The AR group also reported high motivation (mean score: 141.65 ± 19.25) and usability (System Usability Scale score: 90.47 ± 11.91), with low cognitive load (mean score: 15.42 ± 5.76).
Maggio et al., 2022 [20]	Randomized Controlled Trial	16	58.4 years	6 weeks	Cognitive performance (Parkinson’s disease)	Significant cognitive improvement. The GAS score recorded at the end of the study (65.6 ± 4.2) was significantly higher than at baseline (38.5 ± 2.4; *p*-value < 0.001).
Finkelstein et al., 2023 [21]	Pilot Feasibility Study	9	Adults	Pilot period	Pulmonary outcomes	Statistically significant increase in patient knowledge (mean score increase from 7.2 to 7.9; *p* < 0.04), with a high usability score (System Usability Scale: 95.8).
Wells et al., 2023 [22]	Comparative Study	40	Older adults	Study duration	Physical and mental health	Improved physical and psychological health outcomes, statistical significance was not clearly tested.
Lacey et al., 2023 [23]	Randomized Controlled Trial	126	18–64 years	6 weeks	Severity of specific phobias	Significantly greater reduction in specific phobia severity scores (mean change: −20.53 ± 8.24) compared to the waitlist control group (−12.31 ± 10.66), with an effect size of 0.86 (*p* < 0.001).
Chen et al., 2022 [24]	Pilot Study	48	Adults	6 weeks	Functional outcomes post-stroke	Ιmprovements in functional independence (mean FIM score increase of 5.54 points) and quality of life (mean EQ5D-5L score increase of 7.13 points) after a 6-week intervention.
Bruni et al., 2024 [25]	Feasibility Study	34	Older adults (60+)	Single session	Cognitive/memory assessment	Effective cognitive assessment, statistical significance was not clearly tested.
Guldager et al., 2020 [26]	Randomized Controlled Trial	31	Adolescents (16–18)	Single session	Alcohol risk perception	Improved awareness and risk perception, statistical significance was not clearly tested.
Vinci et al., 2020 [27]	Randomized Controlled Trial	50	Adults (average 34)	8 weeks	Smoking abstinence, cravings	Significant craving reduction, improved abstinence. Εxposure to smoking-related augmented reality images elicited a significantly higher urge to smoke (median = 4.58, SD = 3.49) compared to neutral images (median = 1.42, SD = 3.01), with a Wilcoxon signed-rank test yielding Z = −2.14, *p* = 0.03, and a large effect size (Cohen’s d = 0.70).
Martinez-Millana et al., 2024 [28]	Feasibility Study	24	Young adults (18–30)	4 weeks	Physical activity levels	Increased physical activity. Statistical significance was not clearly tested.
Blackmore et al., 2024 [29]	Randomized Controlled Trial	45	Adults (18–60)	8 weeks	Anxiety and depression symptoms	Improved mood, reduced anxiety. Mean curiosity and decentering increased significantly (Cohen d = 1.3 and 1.51, respectively; *p* < 0.001). Negative affect on the Positive and Negative Affect Schedule (Cohen d = 0.62; *p* = 0.003) and State–Trait Anxiety Inventory Y-1 state anxiety (Cohen d = 0.84; *p* < 0.001) significantly reduced.
Ucero-Lozano et al., 2023 [30]	Feasibility Study	14	Adults (30–50)	Single session	Joint mobility, pain management	Improved mobility, reduced pain. Statistically significant differences in joint state (F = 51.38; η^2^p = 0.63), pressure pain threshold of the lateral malleolus (F = 12.34; η^2^p = 0.29), and range of motion (F = 11.7; η^2^p = 0.28).
Tomczyk et al., 2021 [31]	Quasi-experimental 2 × 2 × 2 factorial design	276	Adults (17–83)	Single session	Momentary anxiety	Participants who received a warning message and were confronted with a thunderstorm showed the highest increase in momentary anxiety (video: +0.67, vignette: +0.58; both *p* < 0.001), which predicted information seeking intentions (β = 0.46, *p* < 0.001).
Shen et al., 2024 [32]	Pilot Pre–Post Study	18 Medicaid Providers	Not reported; adult professionals	Single session	Training reaction, affective knowledge, skill-based knowledge	Significant improvement in LGBTQ+ bias-related knowledge (Cohen d = 0.72; 95% CI −1.38 to −0.04).
Klemm et al., 2021 [33]	Feasibility Study	125 (HCPs and medical students)	Adults (18–70)	Single session	Knowledge and attitude changes toward arthritis	Reported improvements in attitudes and empathy, but the statistical significance was not clearly tested.
